# Standardized and hierarchically classified heart failure and complementary disease monitoring outcome measures: European Unified Registries for Heart Care Evaluation and Randomised Trials (EuroHeart)

**DOI:** 10.1093/ehjqcco/qcae086

**Published:** 2024-10-09

**Authors:** Asad Bhatty, Chris Wilkinson, Gorav Batra, Suleman Aktaa, Adam B Smith, Ali Wahab, Sam Chappell, Joakim Alfredsson, David Erlinge, Jorge Ferreira, Ingibjörg J Guðmundsdóttir, þórdís Jóna Hrafnkelsdóttir, Inga Jóna Ingimarsdóttir, Alar Irs, András Jánosi, Zoltán Járai, Manuel Oliveira-Santos, Bogdan A Popescu, Peter Vasko, Dragos Vinereanu, Jonathan Yap, Raffaele Bugiardini, Edina Cenko, Ramesh Nadarajah, Matthew R Sydes, Stefan James, Aldo P Maggioni, Lars Wallentin, Barbara Casadei, Chris P Gale

**Affiliations:** Leeds Institute of Cardiovascular and Metabolic Medicine, University of Leeds, Leeds, UK; Leeds Institute for Data Analytics, University of Leeds, Leeds, UK; Department of Cardiology, Leeds Teaching Hospitals NHS Trust, Leeds, UK; Hull York Medical School, University of York, York, UK; Academic Cardiovascular Unit, South Tees NHS Foundation Trust, James Cook University Hospital, Middlesbrough, UK; Department of Medical Sciences, Cardiology and Uppsala Clinical Research Centre, Uppsala University, Uppsala, Sweden; Leeds Institute of Cardiovascular and Metabolic Medicine, University of Leeds, Leeds, UK; Department of Cardiology, Leeds Teaching Hospitals NHS Trust, Leeds, UK; Leeds Institute of Cardiovascular and Metabolic Medicine, University of Leeds, Leeds, UK; Leeds Institute for Data Analytics, University of Leeds, Leeds, UK; Leeds Institute of Cardiovascular and Metabolic Medicine, University of Leeds, Leeds, UK; Leeds Institute for Data Analytics, University of Leeds, Leeds, UK; Department of Cardiology, Leeds Teaching Hospitals NHS Trust, Leeds, UK; Leeds Institute of Cardiovascular and Metabolic Medicine, University of Leeds, Leeds, UK; Leeds Institute for Data Analytics, University of Leeds, Leeds, UK; Linköping University Hospital, Linköping, Sweden; Lund University, Sweden; Hospital de Santa Cruz, Centro Hospitalar de Lisboa Ocidental, Portugal; Department of Cardiology, Landspitali University Hospital, Reykjavik, Iceland; Department of Health Sciences, Faculty of Medicine, University of Iceland, Reykjavik, Iceland; Department of Cardiology, Landspitali University Hospital, Reykjavik, Iceland; Department of Health Sciences, Faculty of Medicine, University of Iceland, Reykjavik, Iceland; Department of Cardiology, Landspitali University Hospital, Reykjavik, Iceland; Department of Health Sciences, Faculty of Medicine, University of Iceland, Reykjavik, Iceland; Tartu University Hospital, Estonia; György Gottsegen National Cardiovascular Institute, Hungary; South Buda Center Hospital, Szent Imre Teaching Hospital, Hungary; Cardiology Department, Unidade Local de Saúde de Coimbra, Portugal; University of Medicine and Pharmacy Carol Davila, Emergency Institute for Cardiovascular Diseases, Bucharest, Romania; Linköping University Hospital, Linköping, Sweden; University of Medicine and Pharmacy Carol Davila, Emergency Institute for Cardiovascular Diseases, Bucharest, Romania; National Heart Centre Singapore, Singapore; ANMCO Research Centre, Heart Care Foundation, 50121 Florence, Italy; ANMCO Research Centre, Heart Care Foundation, 50121 Florence, Italy; Leeds Institute of Cardiovascular and Metabolic Medicine, University of Leeds, Leeds, UK; Leeds Institute for Data Analytics, University of Leeds, Leeds, UK; Department of Cardiology, Leeds Teaching Hospitals NHS Trust, Leeds, UK; MRC Clinical Trials Unit at UCL, University College London, London, UK; BHF Data Science Centre, Health Data Research UK, London, UK; Data for R&D Programme, NHS England, London, UK; Department of Medical Sciences, Cardiology and Uppsala Clinical Research Centre, Uppsala University, Uppsala, Sweden; ANMCO Research Centre, Heart Care Foundation, 50121 Florence, Italy; Department of Medical Sciences, Cardiology and Uppsala Clinical Research Centre, Uppsala University, Uppsala, Sweden; Division of Cardiovascular Medicine, NIHR Oxford Biomedical Research Centre, University of Oxford, Oxford; Leeds Institute of Cardiovascular and Metabolic Medicine, University of Leeds, Leeds, UK; Leeds Institute for Data Analytics, University of Leeds, Leeds, UK; Department of Cardiology, Leeds Teaching Hospitals NHS Trust, Leeds, UK

**Keywords:** Outcomes definitions, Heart failure, EuroHeart, Quality of care

## Abstract

**Aims:**

The lack of standardized definitions for heart failure outcome measures limits the ability to reliably assess the effectiveness of heart failure therapies. The European Unified Registries for Heart Care Evaluation and Randomised Trials (EuroHeart) aimed to produce a catalogue of internationally endorsed data definitions for heart failure outcome measures.

**Methods and results:**

Following the EuroHeart methods for the development of cardiovascular data standards, a working group was formed of representatives from the European Society of Cardiology Heart Failure Association and other leading heart failure experts. A systematic review of observational and randomized clinical trials identified current outcome measures, which was supplemented by clinical practice guidelines and existing registries for contemporary definitions. A modified Delphi process was employed to gain consensus for variable inclusion and whether collection should be mandatory (Level 1) or optional (Level 2) within EuroHeart. In addition, a set of complementary outcome measures were identified by the working group as of scientific and clinical importance for longitudinal monitoring for people with heart failure. Five Level 1 and two Level 2 outcome measures were selected and defined, alongside five complementary monitoring outcomes for patients with heart failure.

**Conclusion:**

We present a structured, hierarchical catalogue of internationally endorsed heart failure outcome measures. This will facilitate quality improvement, high quality observational research, registry-based trials, and post-market surveillance of medical devices.

## Graphical Abstract

**Graphical Abstract:**
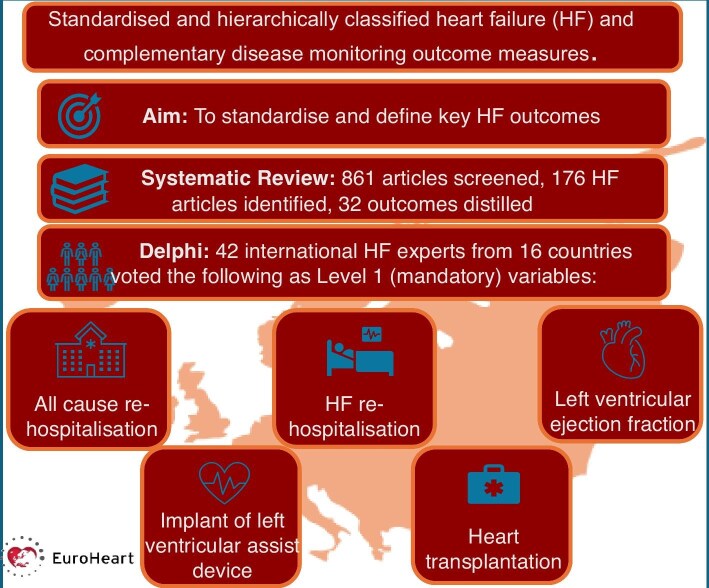
Standardized and hierarchically classified heart failure and complementary disease monitoring outcome measures. HF, heart failure.

Key Learning Points **What is already known**

- Heart failure is a common long-term condition that is associated with significant morbidity and mortality.
- Inconsistent cardiovascular outcome measures and definitions hinder interpretation between trials.
- Existing consensus documents have sought to standardize heart failure outcome measures for use in trials. However, they are not specific to heart failure, address acute decompensated heart failure, or lack hierarchical specification.

**What this study adds**

- A structured and hierarchical catalogue of heart failure outcome measures that are internationally endorsed by ESC affiliations and working groups including the Heart Failure Association.
- These include five Level 1 outcomes (mandatory within EuroHeart), two Level 2 outcomes (optional), and five complementary outcomes (optional).
- Addition of complementary heart failure outcomes that are useful to monitor in patients with heart failure.

## Introduction

Heart failure is a common long-term condition associated with substantial morbidity and mortality, reduced quality of life, and high economic burden.^1–3^ Advances in research study design, generalizability of results, and their translation into clinical practice is contingent upon consistent, clear definitions of clinical outcomes that are widely applicable.^4^ Inconsistent outcome measures and definitions in studies of the same intervention hinders interpretation of the effect of the interventions being tested.^4^ Previous work from the Academic Research Consortium (ARC)^5,6^ and the Standardised Data Collection for Cardiovascular Trials Initiative^7^ sought to standardize outcome measures and their definitions for cardiovascular disease. However, these outcome measures are not specific to contemporary heart failure management,^7^ limited to acute decompensated heart failure,^6^ or lack hierarchical grading of the perceived importance of the outcome measure to health care providers, trialists, and regulators.^5^

The European Unified Registries for Heart Care Evaluation and Randomised Trials (EuroHeart) is a European Society of Cardiology (ESC) initiative that aims to improve the quality of care and outcomes for patients with cardiovascular disease. To achieve this, EuroHeart has published a suite of internationally endorsed data standards for cardiovascular diseases using an established methodology.^8–12^ EuroHeart is prospectively and continuously capturing patient data across participating countries from multiple geographies as part of a collaborative international registry of patients with acute coronary syndrome,^13^ and will now expand to other cardiovascular disease areas including heart failure. EuroHeart will facilitate harmonized country-level quality improvement and will generate the basis for international observational and registry-based randomized controlled trials, and post-marketing surveillance of devices and pharmacotherapies. Robust, internationally agreed, and standardized clinical outcome measures are therefore required.^4^

We aimed to produce a catalogue of hierarchically classified standardized heart failure outcome measures and their definitions in collaboration with the Heart Failure Association (HFA) and other international heart failure experts.

## Methods

### Data Science Group

The Data Science Group comprises of a project chair (CPG), medical experts (CW, AB, GB), project manager (CR), statistician (ABS), and data manager (SC).

### Methodology

We followed the EuroHeart methodology for the development of data standards.^12^ Briefly, this involved: (i) a systematic review of the literature to compose a list of ‘candidate’ outcome measures for heart failure; (ii) the selection and prioritization of variables by domain experts in the working group using a modified Delphi method; and (iii) the synthesis of outcome measure definitions based upon the existing literature, with critical review by the working group.

EuroHeart has already set out ‘generic’ cardiovascular outcomes measures that are applicable to all patients with cardiovascular disease, including heart failure.^14^ These include all-cause mortality, cardiovascular mortality, myocardial infarction, stroke, and new onset heart failure. This article should be considered in concert with these EuroHeart generic outcome measures.

### Systematic review

We performed a systematic review of the literature on primary and secondary outcome measures reported in cardiovascular studies relevant to heart failure published between 1 January 2000 and 7 September 2023. This included peer reviewed randomized clinical trials and observational studies published in highly cited medical journals (Lancet, Journal of the American Medical Association, and New England Journal of Medicine). Definitions from existing heart failure registries, previous consensus documents, and contemporary guidelines were screened,^1,5,7^ with the synthesized results providing the basis for the modified Delphi process undertaken with the working group.

### Working group

A working group was formed to identify clinically relevant outcome measures for the management of heart failure and agree upon their definitions for the variables via virtual meetings and polls. The working group included members of the EuroHeart Data Science Group, representatives from the HFA and ESC working groups (Appendix) as well as external heart failure experts. In total, the working group included 42 experts spanning 16 countries across Europe and North America.

### Modified Delphi process

By means of a poll, each member of the working group independently reviewed the list of outcome measures derived from the literature review and voted to classify them as either a Level 1 (mandatory), Level 2 (optional), or to exclude the variable. This judgement was based upon the respondent's expertise concerning the importance, supporting evidence base, validity, reliability, feasibility, and applicability of each variable.

The threshold for inclusion as a Level 1 variable was at least 75% of participants voting for selection of the variable as Level 1.^15^ The threshold for inclusion as a Level 2 variable was at least 75% of participants selecting for the variable either as Level 1 or Level 2. The results of the poll were presented and discussed among the working group during an online meeting held on 28 October 2023. Participants were invited to provide feedback during voting and there was a proposal made by the experts that additional variables to monitor disease progression and response to therapy over time would be valuable. This resulted in a re-vote on the complementary variables by means of an online poll. The agreed list of variables and their definitions were then reviewed by the working group for ratification.

### Hierarchical grading

The working group classified outcome measures as Level 1 that should be collected for all participants, and Level 2 that may be selected by participating centres depending on their own requirements.

### Implementation and application

The final set of Level 1 heart failure outcome measures will be programmed into the EuroHeart IT system by the EuroHeart Registry Technology Group. Data recorded on the IT platform will have an associated date of outcome occurrence and outcome multiplicity is allowed (except for the occurrence and date of death). The expected target population will be patients hospitalized after an index presentation of heart failure or after an outpatient visit for heart failure at a participating centre in EuroHeart countries. Data reporting and its statistical analysis will be in accordance with a statistical analysis plan.

### Patient involvement

Patients were not invited to the vote on the candidate list of variables as per their request as they advised us that the process was too technical. The results of the poll have been presented to the ESC patient forum, which support this work.

## Results

The systematic review retrieved 4728 studies, of which 861 (18%) met the inclusion criteria. Of these, 176 (20%) studies concerned heart failure. The potentially relevant outcome measures were extracted by members of the Data Science Group and were supplemented by those used in existing registries. In total, 34 candidate outcomes measures for heart failure were presented to the working group for independent voting. The final set of outcome measures was selected after a series of meetings and online polls between 16 July and 28 October 2023.

### Hierarchical outcomes

#### Level 1 (mandatory) outcome measures

There were five outcome measures specific to heart failure that were deemed mandatory to collect and defined as Level 1 by the working group. These were in addition to the EuroHeart generic Level 1 cardiovascular outcome measures.

**Figure 1:**
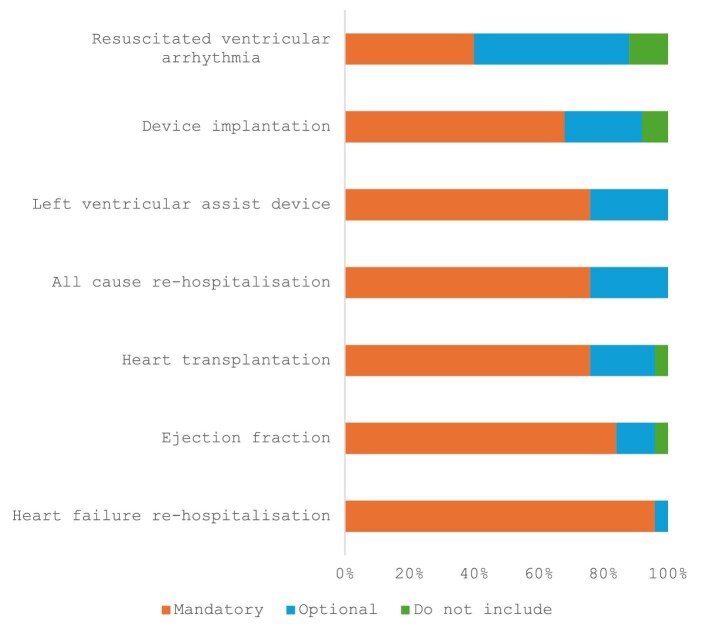
Distribution of votes for Level 1 and Level 2 heart failure outcome measures. Level 1 variable: Mandatory; Level 2 variable: Optional.A total of twenty-five international heart failure experts voted on the above outcome measures. Device implantation refers to leadless, single and dual chamber pacemakers, subcutaneous, extravascular and subcutaneous defibrillators and cardiac resynchronisation therapies.

These were: (i) capture of left ventricular ejection fraction as a percentage. Where this is not possible, the category reported should be according to ESC guidance,^16^ (ii) all-cause hospitalization, (iii) heart failure hospitalization, (iv) implantation of left ventricular assist device, and (v) heart transplantation (*Figure 1* and *Table 1*).

**Table 1:** Level 1 and 2 heart failure outcome measures and definitions



Heart failure: Level 1 variables	
All-cause rehospitalization	Unscheduled hospitalization for any cause, defined as a being admitted for more than 24 h or past a calendar day.^a,b^
Heart failure rehospitalization	Hospital admission primarily related to heart failure (HF). Heart failure is a clinical syndrome characterized by typical symptoms (e.g. dyspnoea) and/or signs (e.g. ankle swelling), caused by a structural and/or functional cardiac abnormality (e.g. left ventricular hypertrophy or impairment), and associated with elevated natriuretic peptide levels and/or objective evidence of pulmonary or systemic congestion from a cardiogenic origin at rest or with exercise. Unplanned HF hospitalization is defined as a patient requiring an unscheduled hospital admission for a *primary diagnosis* of HF with a length of stay that either exceeds 24 h or crosses a calendar day (if hospital admission and discharge times are unavailable). To satisfy the criteria for a HF hospitalization, the patient must be admitted primarily for HF with signs, symptoms, and diagnostic testing results identical to those already described above. The patient must also require treatment for HF such as significant augmentation of oral diuretics, intravenous diuretics, or mechanical or surgical intervention for HF.^a–d^
Left ventricular ejection fraction	Let ventricular ejection fraction, ideally measured with echocardiography.
Heart transplantation	Receipt of surgery in which a failing, diseased heart is replaced with a healthier donor heart.^e^
Left ventricular assist device	Implant of a left ventricular assist device (LVAD).
**Heart failure: level 2 variables**	
Device implantation	Implantation of: • Transvenous permanent pacemaker is an electronic device that is implanted in the subcutaneous tissue and gives the heart an electrical stimulation through transvenous wires. • Leadless pacemaker is an electronic device that is implanted directly into the right ventricle. • Transvenous implantable cardioverter defibrillator (ICD) is a device that is used to correct abnormal heartbeat through transvenous wires. • Subcutaneous ICD is an ICD with a pre-sternal lead and is positioned between the latissimus dorsi and serratus muscle within the subcutaneous tissue. • Extravascular ICD is an ICD with a substernal lead and the device in the subcutaneous tissue of the lateral thorax. • Cardiac resynchronization therapy (CRT) device and pacemaker (CRT-P) is defined as a biventricular pacemaker that sends electrical stimulation to both ventricles. • CRT-D is a biventricular pacemaker and defibrillator.^f,g^
Resuscitated ventricular tachyarrhythmia	The patient was successfully resuscitated and had return of spontaneous circulation from a ventricular tachyarrhythmia.
NYHA class	NYHA class I: no limitations of physical activity. Ordinary physical activity does not cause undue fatigue, palpitations, or dyspnoea. NYHA class II: slight limitation of physical activity. The patient is comfortable at rest. Ordinary physical activity results in fatigue, palpitations, or dyspnoea. NYHA class III: marked limitation of physical activity. The patient is comfortable at rest. Less than ordinary activity causes fatigue, palpitations, or dyspnoea. NYHA class IV: inability to carry on any physical activity without discomfort. Heart failure symptoms are present even at rest or with minimal exertion.^c,h^
Change in left ventricular ejection fraction	Left ventricular ejection fraction, ideally measured with echocardiography.
Atrial fibrillation	Patient has a concurrent diagnosis of any type of atrial fibrillation with heart failure. Atrial fibrillation is defined as a supraventricular tachyarrhythmia with uncoordinated atrial electrical activation and consequently ineffective atrial contraction. The minimum duration of an ECG tracing of atrial fibrillation required to establish the diagnosis of clinical atrial fibrillation is at least 30 s, or the entire 12-lead ECG. Atrial flutter is defined as a supraventricular tachyarrhythmia with coordinated but overly rapid atrial electrical activation, usually with some degree of atrioventricular (AV) node conduction block. The minimum duration of an ECG tracing of atrial flutter required to establish the diagnosis of clinical atrial flutter is at least 30 s, or the entire 12-lead ECG.^i^
NT-proBNP (ng/L)	Serum NT-proBNP in ng/L.
Estimated glomerular filtration rate (eGFR) (mL/min/1.73 m^2^)	Estimated glomerular filtration rate (eGFR) in mL/min/1.73 m^2^.


References

^a^Généreux P, Piazza N, Alu MC*, et al.* Valve Academic Research Consortium 3: updated endpoint definitions for aortic valve clinical research. *Eur Heart J* 2021;**42**:1825–1857. doi: 10.1093/eurheartj/ehaa799

^b^Hicks KA, Mahaffey KW, Mehran R*, et al.* 2017 Cardiovascular and stroke endpoint definitions for clinical trials. *Circulation* 2018;**137**:961–972. doi: 10.1161/circulationaha.117.033502

^c^Bozkurt B, Coats AJ, Tsutsui H*, et al.* Universal definition and classification of heart failure: a report of the Heart Failure Society of America, Heart Failure Association of the European Society of Cardiology, Japanese Heart Failure Society and Writing Committee of the Universal Definition of Heart Failure. *J Card Fail* 2021. doi: 10.1016/j.cardfail.2021.01.022

^d^McDonagh TA, Metra M, Adamo M*, et al.* 2023 Focused update of the 2021 ESC Guidelines for the diagnosis and treatment of acute and chronic heart failure: developed by the task force for the diagnosis and treatment of acute and chronic heart failure of the European Society of Cardiology (ESC) with the special contribution of the Heart Failure Association (HFA) of the ESC. *Eur Heart J* 2023;**44**:3627–3639. doi: 10.1093/eurheartj/ehad195

^e^Mehra MR, Canter CE, Hannan MM*, et al.* The 2016 International Society for Heart Lung Transplantation listing criteria for heart transplantation: a 10-year update. *J Heart Lung Transplant* 2016;**35**:1–23. doi: 10.1016/j.healun.2015.10.023

^f^Glikson M, Nielsen JC, Kronborg MB*, et al.* 2021 ESC Guidelines on cardiac pacing and cardiac resynchronization therapy. *Eur Heart J* 2021;**42**:3427–3520. doi: 10.1093/eurheartj/ehab364

^g^Friedman P, Murgatroyd F, Boersma LVA*, et al.* Efficacy and safety of an extravascular implantable cardioverter-defibrillator. *N Engl J Med* 2022;**387**:1292–1302. doi: 10.1056/NEJMoa2206485

^h^McDonagh TA, Metra M, Adamo M*, et al.* 2021 ESC Guidelines for the diagnosis and treatment of acute and chronic heart failure. *Eur Heart J* 2021;**42**:3599–3726. doi: 10.1093/eurheartj/ehab368

^i^Hindricks G, Potpara T, Dagres N*, et al.* 2020 ESC Guidelines for the diagnosis and management of atrial fibrillation developed in collaboration with the European Association for Cardio-Thoracic Surgery (EACTS): The Task Force for the diagnosis and management of atrial fibrillation of the European Society of Cardiology (ESC) Developed with the special contribution of the European Heart Rhythm Association (EHRA) of the ESC. *Eur Heart J* 2021;**42**:373–498. doi: 10.1093/eurheartj/ehaa612

#### Level 2 (optional) outcome measures

There were two outcome measures that were deemed optional to collect and defined as Level 2 by the working group (*Figure 1* and *Table 1*).

These were device implantation that included: transvenous pacemakers; leadless pacemakers; transvenous; subcutaneous implantable cardioverter defibrillators; implantable cardioverter defibrillators; cardiac resynchronization therapy—pacemaker; and cardiac resynchronization therapy—defibrillator and resuscitated ventricular arrhythmia.

### Complementary monitoring outcome measures

**Figure 2:**
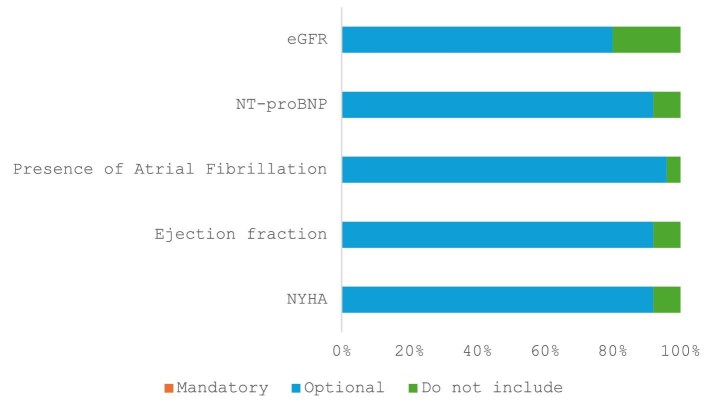
Distribution of votes for the heart failure complementary outcomes. A total of twenty-five international heart failure experts voted on the above outcome measures.

The working group proposed additional heart failure outcome measures that may be used for monitoring patients with heart failure, supplementary to the Level 1 and Level 2 heart failure outcome measures. These were: (i) concurrent presence of atrial fibrillation (AF), classified as first diagnosed AF, paroxysmal, persistent or permanent as defined by ESC guidelines^17^); (ii) N-terminal brain natriuretic peptide levels (NT-proBNP); (iii) estimated glomerular filtration rate (eGFR); (iv) change in left ventricular ejection fraction [i.e. the difference in the left ventricular ejection fraction (%) be measurement using the same imaging modality for calculating left ventricular ejection fraction]; and (v) New York Heart Association class (NYHA) (*Figure 2* and *Table 1*).

## Discussion

Through a structured and collaborative international expert-led process, we have identified and defined a catalogue of hierarchical outcome measures for patients with heart failure, including a complementary suite of monitoring variables. These will be used to measure the clinical outcomes for participants in EuroHeart and have wider utility for randomized clinical trials, prospective observational cohorts, and clinical registries outside EuroHeart.

The identification and optimal clinical management of heart failure is critical, given the increasing prevalence of heart failure and represents a significant health burden across Europe.^3^ Recent advances in guideline directed care have been associated with improved symptoms, better quality of life, reduced all-cause mortality, and fewer heart failure readmissions.^18,19^ Nonetheless, translating clinical guidelines into real world practice can be challenging. Indeed, previous work has shown that provision of guideline directed care for heart failure is variable between and within the European countries and this can be associated with adverse outcomes.^20,21^ One possible cause for this could be variability in defining key outcome measures^4^ which can impact heart failure hospitalization rates.^22^

The EuroHeart heart failure outcome measures build upon existing cardiovascular outcomes relevant to patients with heart failure, including those by the ARC.^5,7^ There are similarities between these outcomes set: like ARC, all-cause, and cardiovascular-specific mortality were included as mandatory variables within EuroHeart. These are important safety outcomes that are necessary for regulatory approval of device and pharmacological interventions within cardiology.^1^ Similarly, all-cause and heart failure hospitalizations were included, and are predictors of mortality and disease severity.^23^ They are also important for patients,^23^ and health services,^20^ and in research are often components of a composite outcome.^24^ Left ventricular assist device and heart transplantation likewise was included within both EuroHeart and ARC given their importance in advanced disease management and increasing within Europe.^1,2^

Both organizations provide similar definitions for heart failure hospitalization, with emphasis on the admission to hospital being attributed primarily to heart failure and that the hospitalization must exceed 24 h or cross a calendar day. For worsening heart failure to be defined as an outcome measure, both organizations agree upon the requisite for clinical, biomarker and radiological markers, and augmentation of medical therapy from baseline.

Categorizing and defining heart failure outcomes in hierarchical fashion is a hallmark feature of EuroHeart data standards,^8–11^ which differs from previous work on heart failure outcomes set out by the ARC.^5^ Previous studies on heart failure have graded outcomes hierarchically based on their importance to both clinicians and regulators, which reflects an ambition from researchers and regulators to adopt a more pragmatic approach to analysis within research.^25,26^

In contrast to ARC, recording left ventricular ejection fraction is a Level 1 outcome in EuroHeart. Current guidelines stratify heart failure according to left ventricular ejection fraction categories due to differences in the benefit of heart failure therapies and the association of worsening outcomes with declining left ventricular ejection fraction.^16,21^ However, heart failure with recovery or improvement in ejection fraction is increasingly recognized after implementing guideline-directed therapy and is associated with better long term outcomes.^27^ Therefore, the working group agreed that left ventricular ejection fraction should be included both as a stand-alone variable and a variable that can be used for monitoring heart failure.

We also define complementary outcomes that may be used for the longitudinal evaluation of patients with heart failure, beyond traditional ‘hard’ outcomes.^28^ These variables are either mechanistic or surrogate outcomes that, if collected prospectively could form the basis of further research. For example, the Valsartan Heart Failure Trial (Val-HeFT) investigated left ventricular ejection fraction as a surrogate outcome in patients with an ejection fraction below 35% that were randomized to valsartan or placebo. Compared to placebo, patients taking valsartan demonstrated an improvement in left ventricular ejection fraction, improved survival at 12 months and decreased NT-proBNP level.^27^ Given the advances in optimal medical therapy in heart failure, monitoring the left ventricular ejection fraction as well as other complementary variables could form the basis of observational research in real world settings. The working group emphasized the need for consistency in the method of measurement, for example serial echocardiogram scans.

The relationship between atrial fibrillation and heart failure is complex, because AF can be either the cause or consequence of heart failure.^29^ Studies have shown that catheter ablation in patients with symptomatic paroxysmal or persistent AF in the context of severe left ventricular systolic dysfunction improves outcomes compared to medical therapy alone.^30,31^ This highlights the importance of recognizing and considering AF as a potentially therapeutic target in heart failure in contemporary registries or a surrogate marker for deterioration in heart failure.^32^ Other outcomes such as eGFR was included given renal impairment is well known to be greatly associated with mortality in patients with heart failure^33^ and its prominence as a renal outcome in contemporary heart failure trials.^34,35^

EuroHeart aims to reduce the burden of cardiovascular disease across Europe. The publication of these variables and their definitions will allow us to better understand the outcomes for people with heart failure. They will be integrated with the existing registries,^8–11^ and incorporated into the EuroHeart IT platform. This will allow the patterns of clinical care and outcomes of patients to be evaluated longitudinally across Europe, and provide a platform for international quality improvement to address any unwarranted variation in care,^3,16^ and facilitate quality benchmarking.^36^ It is therefore important to health care providers, funders, and patients to integrate and optimize provision of guideline-indicated care, whilst monitoring outcomes for patients. By harmonizing data collection from distinct European heart failure registries into an international collaboration together, EuroHeart can better inform and improve cardiovascular care continuously.^37^ Integral to this process, however, is the adoption of a catalogue of standardized definitions of cardiovascular outcome measures that allows for research to be more externally generalizable and potentially more efficient in its delivery.^4,37^

We used a robust methodology and harnessed the expertise of a wide range of international experts in heart failure to identify and define these variables. However, we recognize the limitations of this work. Although the outcome measures and their definitions were distilled from a systematic review, the final selection of outcomes were agreed upon by consensus of the international experts within the working group and are therefore subject to selection bias. Nevertheless, the experts that composed the working group were taken from a broad range of countries with a wealth of experience and knowledge within registry work and trials. Furthermore, these definitions were endorsed by the ESC Patient Forum as well as the ESC Committee for Young Cardiovascular Professional that provided an additional layer of validity in our findings. Although the importance of patient reported outcomes measures (PROMs) and experiences (PREMs) is increasingly recognised,^38,39^ our remit for this project was limited to clinical outcomes, which was decided by international consensus of the working group. A further project involving PROMs use within heart failure and other cardiovascular conditions is anticipated.

## Conclusion

This document provides a structured, hierarchical catalogue of heart failure outcome measures that are internationally endorsed that includes complementary outcomes. For EuroHeart, this will facilitate quality improvement, prospective observational research, randomized clinical trials and post-market surveillance of medical devices across Europe.

## Notes

Consolidation phase (since October 2022) industry partners: AstraZeneca AB; Boehringer Ingelheim; Novartis Pharma AG; Roche; Swedish Heart Lung Foundation; and Bayer AG.

## Notes

**Conflict of interest:** There are no declarations with respect to this manuscript. A.B., A.B.S., A.W., S.C., D.E., E.C., I.J.G., þ.J.H., I.J.I., A.J., P.V., R.B., and L.W. report no conflicts of interest. C.W. reports grants from British Heart Foundation and National Institute for Health Research and has unpaid roles with EHJ Quality of Care and Clinical Outcomes (Associate Editor), and NICE Indicator Advisory Committee. G.B., outside the submitted work, received institutional grants from Bayer, P.O. Zetterlings Foundation, Jeanssons Foundation, AstraZeneca, and Pfizer and honoraria for lectures from AstraZeneca, Bristol-Myers Squibb, Boehringer Ingelheim, Novo Nordisk, Pfizer, and Sanofi. S.A. reports participation on a Data Safety Monitoring Board or Advisory Board for the British Coronary Intervention Society (BCIS) and leadership or fiduciary role in European Society of Cardiology quality of care programme. J.A. has received lecture fees from AstraZeneca, Bayer, Boehringer Ingelheim, MSD, and Novartis with advisory board re-imbursements for AstraZeneca and Novartis. J.F. has received consulting fees for Boehringer Ingelheim, lecture fees from Amgen and Bayer and Participation on a Data Safety Monitoring Board or Advisory Board for Boehringer Ingelheim, Merck, Sharp & Dohme, Novartis and is on the steering committee for VESALIUS-CV, ESSENCE CS9, OCEAN(a) Outcomes, LIBREXIA-AF, and VICTORION-2-Prevent. A.I. has received grants from Abbott, Novartis, and Medtronic, support for attending meetings from Abbott and Medtronic, is a board member (secretary) for the Estonian Society of Cardiology and is a part time employee of the Estonian Agency of Medicines. Z.J. has received consulting fees from AstraZeneca, Novo Nordisk, Gedeon Richter; lecture fees from AstraZeneca, Bayer, Berlin Chemie, Boehringer Ingelheim, Egis, KRKA, Merck, Novartis, NovoNordisk, Pfizer, Richter Gedeon, Sandoz, TEVA and is the president of the Hungarian Society of Hypertension, Secretary of the Hungarian Society of Cardiology and Council Member of the European Society of Hypertension. M.O.S. has received speaker's fees from Abbott, Bayer, Biotronik, Boston Scientific, Daichii Sankyo, and Edwards. B.A.P. has received speaker's fees and research support from GE Healthcare and Hitachi-Aloka. D.V. has received grants from Amgen, Bayer, Celecor, Novartis, and NovoNordisk with consulting fees from Boehringer Ingelheim and Sanofi and speaker's fees from Bayer, Pfizer, and Servier. J.Y. has received a grant from Singhealth Cardiovascular ACP Grant, received speaker's honorarium from Abbott, Biosensors, Biotronik, Boston Scientific, Edwards, GE healthcare, J&J, Kaneka, Medtronic, and Terumo and reports a patent planned for ECG and PCG Monitoring System for Detection of Heart Anomaly and is a treasurer for Singapore Cardiac Society. R.N. has received a grant from the British Heart Foundation, Leeds Hospital Charity, Daiichi Sankyo, British Heart Foundation Clinical Research Collaborative, Health Data Research UK, National Institute of Health and Care Research as well as consulting fees from Vitacam. M.R.S. has received research grants from Astellas, Clovis Oncology, Janssen, Pfizer, Novartis, sanofi-aventis with speaker's fees from Janssen and Eisai and consulting fees from Eli Lilly and is an independent unpaid member of many data monitoring committees from academic sponsors. S.J. has received grants from AstraZeneca, Amgen and Elixir, received institutional consulting fees from Novartis, Novo Nordisk, MSD, and Medtronic with lectures fees from Pfizer and Boehringer Ingelheim. S.J. is also part of the advisory board for New Amsterdam. A.P.M., outside of present work, reports fees for participation on a data safety monitoring board or advisory board from Bayer; paid or unpaid leadership or fiduciary role in other board, society, committee, or advocacy group from AstraZeneca, Novartis, and Sanofi. L.W. is co-chair of the Executive Committee of EuroHeart that is not renumerated. B.C. is co-chair of the Executive Committee of EuroHeart that is not renumerated. C.P.G. has received grants from Alan Turing Institute, British Heart Foundation, National Institute for Health Research, Horizon 2020, Abbott Diabetes, Bristol-Myers Squibb, and European Society of Cardiology and reports consulting fees from AI Nexus, AstraZeneca, Amgen, Bayer, Bristol-Myers Squibb, Boehrinher Ingleheim, CardioMatics, Chiesi, Daiichi Sankyo, GPRI Research B.V., Menarini, Novartis, iRhythm, Organon, and The Phoenix Group and speaker's fees from AstraZeneca, Boston Scientific, Menarini, Novartis, Raisio Group, Wondr Medical, Zydus. C.P.G. is also a deputy editor: EHJ Quality of Care and Clinical Outcomes, NICE Indicator Advisory Committee member, Chair ESC Quality Indicator Committee member, and participated on a Data Safety Monitoring Board or Advisory Board for DANBLCOK trial and TARGET CTCA trial. A.G.A., G.M.C.R., B.G., C.G-T, C.P., C.C., I.U., M.Z., M.C., and O.C. all report no conflicts of interest. A.B.-G., reports lectures fees and/or participated in advisory boards for Abbott, AstraZeneca, Bayer, Boehringer Ingelheim, Medtronic, Novartis, Novo Nordisk, Roche Diagnostics, and Vifor. D.A. reports grant support (to the Institution): BMS/Pfizer, Medtronic, Bayer, Roche-Diagnostics and speaker fees from Amgen, Amarin, AstraZeneca, Bayer, Boehringer Ingelheim, BMS, MSD, Novartis, NovoNordisk, Pfizer, Pharmacosmos, Philips, Roche-Diagnostics, Sanofi, Takeda, and Vifor. G.G. reports institutional grants from Win Medica, Galenica, Pfizer, AstraZeneca, Boehringer Ingelheim, Menarini, Roche Diagnostics and payment or honoraria for lectures, presentations, speakers bureaus, manuscript writing or educational events from Pfizer, Roche Diagnostics, Bayer, MSD, AstraZeneca, Boehringer Ingelheim, Janssen, Bausch Health, Novartis, Menarini, Genesis Pharma, Sanofi, ELPEN Pharmaceuticals, Amgen, Gilead Sciences, VIANEX, Servier, Ferrer, Gossamer Bio. G.G. also reports participation on a Data Safety Monitoring Board or Advisory Board for Boehringer Ingelheim, Janssen, MSD, AstraZeneca, GSK, Sanofi, Amgen, Roche Diagnostics, Bayer, Novartis, and Bausch Health. G.S. reports grants from Vifor Pharma, Novartis, Boehringer Ingelheim, Boston Scientific, AstraZeneca, Pharmacosmos, Merck, Bayer, Cytokinetics and Horizon 2022 funding, consulting fees from TEVA, MIUR (Ministero dell'Istruzione, Universita´ e Ricerca), Medical Education Global Solutions, Atheneum, Genesis, CSL Vifor, Agence Recherche (ANR), AstraZeneca, Translational Medicine Academy Foundation (TMA), Servier and speakers fees for Novartis, Roche, Cytokinetics, Translational Medicine Academy Foundation (TMA), Medtronic, Medical Education Global Solutions, Dynamicom Education, AstraZeneca, Vifor Pharma, Abbott, INTAS, and Pharmacosmos. They are also a board member for the following: AstraZeneca, Edwards LifeScience, Uppsala Clinical Research Center (UCR), CSL Vifor, and Servier. G.F. reports a grant from NIH, consulting fees from Merck, Cytokinetics, AstraZeneca, Abbott, Medtronic, Novartis, Janssen, Amgen, Pfizer and is part of the advisory board for Pfizer. J.A.U. has received institutional research grants from Bayer, Boehringer Ingelheim, Janssen, Sanofi, consulting fees from Boehringer Ingelheim, Novartis, Novavax, Novo Nordisk, Sanofi and speakers fees from Amgen, Astrazeneca, Boehringer Ingelheim, Eli Lilly, GlaxoSmithKline, and Sanofi. L.L. reports research grants from the Swiss Heart Foundation, royalties from XBiotech, international patent, patents planned, issued or pending for Coinventor on the International Patent WO/2020/226993 filed in April 2020; the patent relates to the use of antibodies which specifically bind interleukin-1a to reduce various sequelae of ischaemia-reperfusion injury to the central nervous system and is a counsellor of the European Society for Clinical Investigation and a nucleus Member of the ESC WG in Atherosclerosis and Vascular Biology. M.T.J. is chair-elect, WG eCardiology, ESC CEO, Steno Diabetes Center Copenhagen. M.L. reports research grants from Novartis, AstraZeneca, Novo Nordisk, Boehringer Ingelheim, Bayer and Servier; consulting fees from Novo Nordisk, Novartis, Bayer, AstraZeneca, Boehringer Ingelheim and Servier; speakers fees from Novo Nordisk, AstraZeneca, Novartis, Boehringer Ingelheim, Bayer, and Servier. M.M. reports speakers fees from Abbott Structural Heart, Bayer, Cytokinetics, Boehringer Ingelheim, NovoNordisk, and Roche diagnostics. P.P.R. reports speakers fees from Novartis, Boehringer Ingelheim, Pfizer, AstraZeneca, Vifor, Zoll and participation on a Data Safety Monitoring Board or Advisory Board for Novartis, and Boehringer Ingelheim. J.E. reports research grants from Amgen, American Regent, Bayer, Merck, Otsuka, Novo Nordisk, Applied Therapeutics, Carduirion, CSL-Vifor, AstraZeneca, and BI-Lilly. R.L. reports grants from Medtronic and Livanova; speaker's fees from Medtronic and Xenios and participation on a Data Safety Monitoring Board or Advisory Board for Eurosets and Xenios. R.T. reports speakers fees from Boston Scientific and Pfizer and is a Nucleus member ESC eCardiology Working Group and Ordinary member Dutch Society of Cardiology eHealth & AI working group. R.R.J.v.K. reports grants from Dutch Heart Foundation and Hartekind; speaker's fees from Novartis and Bayer and is a member of the ESC working group on Aorta & Peripheral Artery Disease. S.S. reports grants from DFG and the German Research Foundation; consulting fees from AstraZeneca, Novartis, Bristol-Myers Squibb, Boehringer Ingelheim, Lilly; speaker's fees from AstraZeneca, Novartis, Berlin-Chemie, Bristol-Myers Squibb, Boehringer Ingelheim, Lilly and was part of the advisory board for the CASTLE-HTX-Trial. S.M-E is a EACVI Board Member, EACVI Chair of Web and Comm—Unpaid and ESC Communication Committee Member-Unpaid. S.J.G. reports grants from Novartis, Sanofi, Pfizer, AstraZeneca, Cytokinetics, Merck, Boehringer Ingelheim; consulting fees from Amgen, AstraZeneca, Bayer, Bristol-Myers Squibb, Cytokinetics, Boehringer Ingelheim, PharmaIN, Urovant Pharmaceuticals, Eli Lilly, Merck, Sanofi, Tricog Health, Corteria Therapeutics, Roche Diagnostics and speaker's fees from Boehringer Ingelheim, Cytokinetics, Lexicon, Bayer, and Roche Diagnostics.
